# Bioprosthetic Valve Fracturing: *In vitro* Testing of Edwards PERIMOUNT Model P 2900

**DOI:** 10.3389/fcvm.2022.859088

**Published:** 2022-06-02

**Authors:** Hendrik Ruge, Hector A. Alvarez-Covarrubias, Oliver Deutsch, Zahra Alalawi, Keti Vitanova, Rüdiger Lange

**Affiliations:** ^1^Department of Cardiovascular Surgery, German Heart Center, INSURE (Institute for Translational Cardiac Surgery), TUM, Munich, Germany; ^2^German Heart Center Munich, Department of Cardiovascular Surgery, TUM, Munich, Germany; ^3^German Heart Center Munich, Department of Cardiology, TUM, Munich, Germany; ^4^Centro Medico Nacional Siglo XXI, Hospital de Cardiologia, IMSS, Ciudad de México, Mexico

**Keywords:** bioprosthetic valve fracturing, valve-in-valve transcatheter aortic valve replacement, *in-vitro*, balloon rupture, transprosthetic gradient

## Abstract

**Background:**

Bioprosthetic valve fracturing (BVF) results in low gradients following valve-in-valve transcatheter aortic valve replacement (ViV-TAVR). For the commonly used Edwards PERIMOUNT valve data from bench-testing are lacking to provide technical specifications for successful BVF during ViV-TAVR.

**Methods:**

Using four Perimount 19- and 21-mm valves, *in-vitro* high-pressure balloon valvuloplasty with the True Dilatation Balloon Valvuloplasty Catheter and Atlas Gold PTA Dilatation Catheter was performed to analyze balloon-oversizing and pressure-thresholds to successfully achieve BVF.

**Results:**

High-pressure balloons one millimeter larger than the labeled valve size and pressure rates of 20 atm (for Perimount 19-mm) and > 22 atm (for Perimount 21-mm) were required to achieve BVF. Caliper measurements demonstrated 2.5 mm (Perimount 19-mm) and 1.5 mm (Perimount 21-mm) enlarged inner prosthetic diameters after BVF. The Atlas TM Gold PTA Dilatation Catheter achieved BVF with the Perimount 21-mm, whereas the True TM Dilatation Balloon Valvuloplasty Catheter failed in the Perimount 21-mm either for balloon-rupture or pinhole-defect.

**Conclusion:**

Both 19-mm and 21-mm Perimount P 2900 are amendable to BVF, thereby increasing the inner prosthetic diameter. High-pressure balloons 1 mm larger than the labeled valves are essential for this purpose, and the Atlas Gold PTA Dilatation Catheter alone should ensure success in the 21-mm prosthetics.

## Introduction

Valve-in-valve transcatheter aortic valve replacement (ViV-TAVR) is an established therapy for failing surgical bioprostheses in patients with higher operative risks (1, 2). One-year survival after ViV-TAVR is 83%, but mean transprosthetic pressure gradients are determined by the size of bioprosthetics previously implanted (2). In a cohort with small-sized surgical valves (<21 mm inner prosthetic diameter), higher gradients and poor 8-year survival have resulted from ViV-TAVR procedures (1). Bioprosthetic valve fracturing (BVF) is intended to lower transprosthetic gradients in this setting, especially in patients with small surgical valves (3–5). Although technical specifications (ie, balloon types, sizes, and pressure ratings) needed for successful BVF have been documented for various surgical valves through *in vitro* testing (6–8), there is limited clinical data on BVF utilization frequency or success rates (4, 9). PERIMOUNT surgical valves (Edwards Lifesciences, Irvine, CA, USA), models 2800 and 2900 in particular, have demonstrated inconsistent BVF success (9). Data from *in vitro* studies of Magna and Magna Ease valves (Edwards Lifesciences) have served to guide clinicians in terms of balloon type and sizing, enabling successful BVF during ViV-TAVR procedures (6). However, the PERIMOUNT model 2700 is known for its resistance to BVF.

Between 2012 and 2018, Edwards PERIMOUNT valves (models 2800 and 2900) have been commonly deployed surgical valves, used in 15,000–20,000 implantations annually throughout Europe and the US (personal communication with Edwards Lifesciences). The present *in vitro* study was undertaken to better understand the amenability of a model 2900 PERIMOUNT valve to BVF attempts, determining pressure rates and balloon oversizing metrics required for successful BVF implementation.

## Methods

### Materials

The Edwards PERIMOUNT (model P2900) 19- and 21-mm valves used for study came from institutional stock. The P2900 valve has three pericardial leaflets mounted on a cobalt-chromium-nickel alloy stent frame. Its sewing ring has a silicone rubber core and a polytetrafluoroethylene (PTFE) skirt.

For *in vitro* BVF testing, two distinctly different high-pressure balloons were engaged: (1) the True Dilatation Balloon Valvuloplasty Catheter (Bard Peripheral Vascular Inc, Temp, AZ, USA), burst-pressure rating of 6 atm, and (2) the Atlas Gold PTA Dilatation Catheter (Bard Peripheral Vascular Inc), burst-pressure rating of 14 atm. The Edwards Inflation Device (Edwards Lifesciences) allows inflation pressures up to 30 atm.

### Bioprosthetic Valve Fracturing

**Figure 1:**
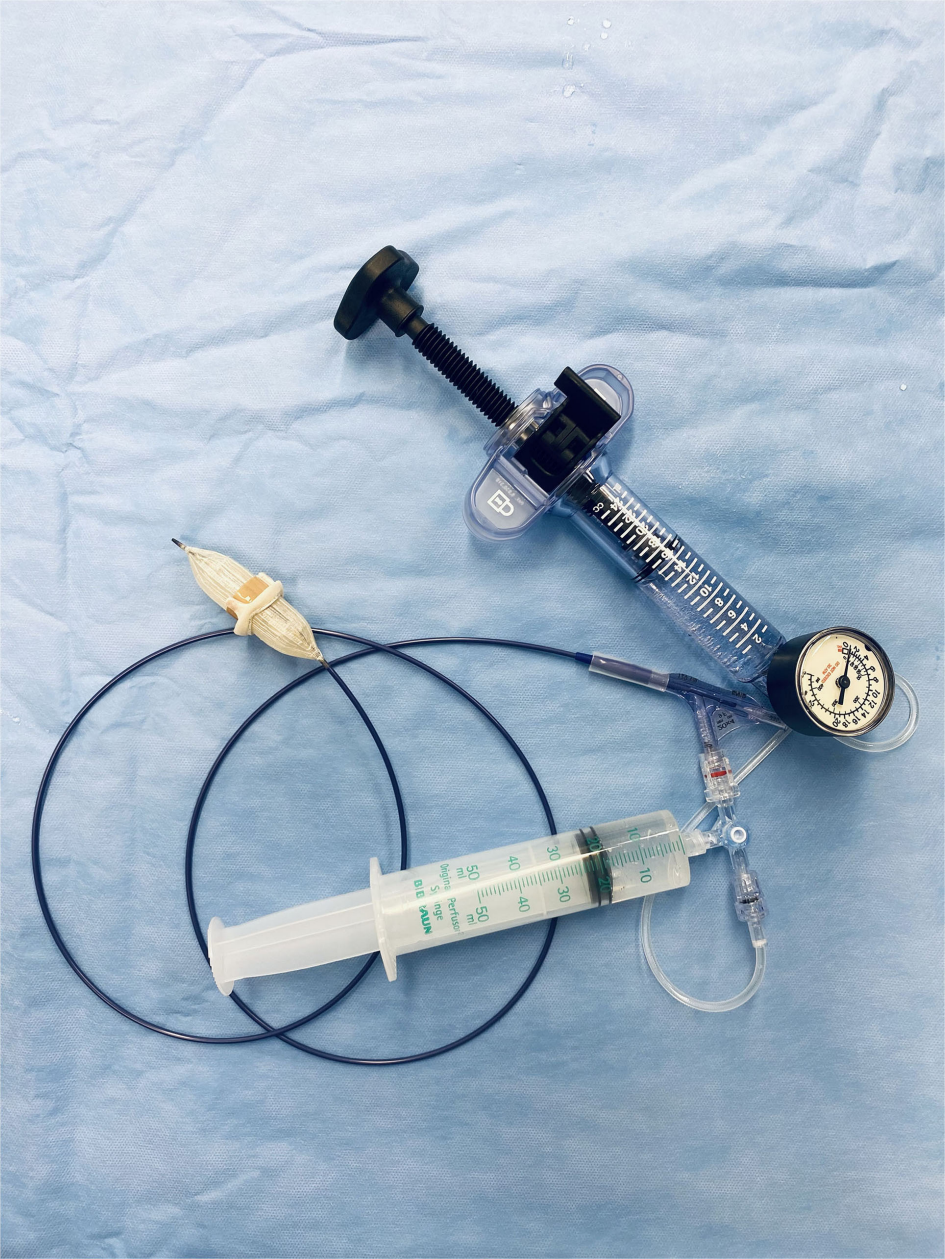
*In-vitro* test setting with a high-pressure balloon connected via high-pressure stopcock to a 50-ml syringe containing dilute contrast and to a high-pressure indeflator.

Balloons for BVF were sized 1–3 mm beyond true inner diameters of surgical valves, using the Valve in Valve App (UBQO Ltd. [London, UK] and Dr. Vinayak Bapat [Minneapolis, MN, USA]). True inner diameters were obtained before and after BVF by caliper. In the course of BVF, a high-pressure balloon was connected via high-pressure stopcock (Marquis; Merit Medical Systems Inc., South Jordan, UT, USA) to a 50-ml syringe containing dilute contrast and to a high-pressure indeflator (Figure 1). Once the balloon filled with contrast, the stopcock was turned, allowing incremental indeflator pressurization to the point of valve fracture or balloon rupture (Supplementary Video 1). The corresponding pressure level was then recorded.

**Figure 2:**
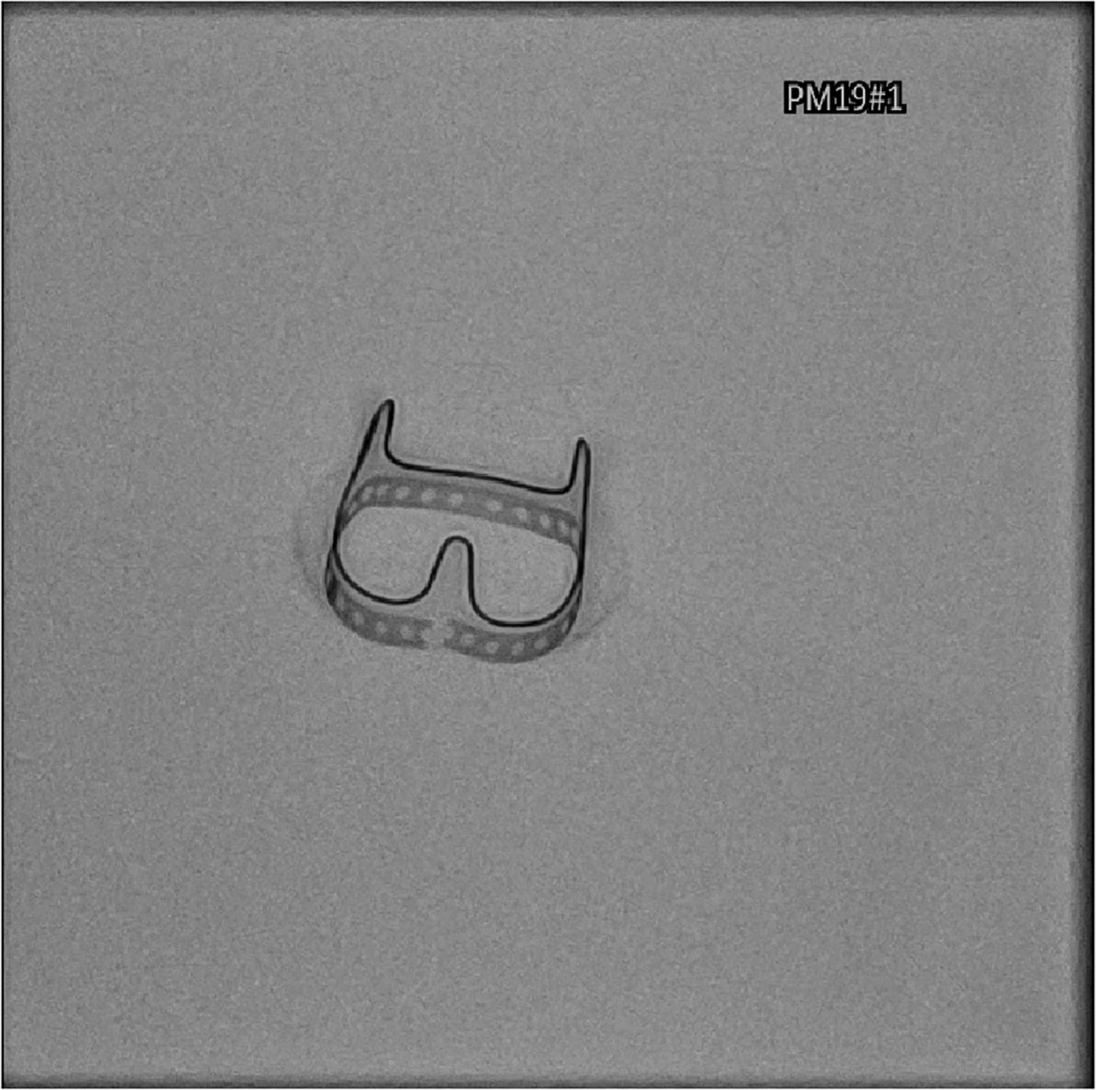
Fluoroscopic confirmation of successful bioprosthetic valve fracturing.

**Figure 3:**
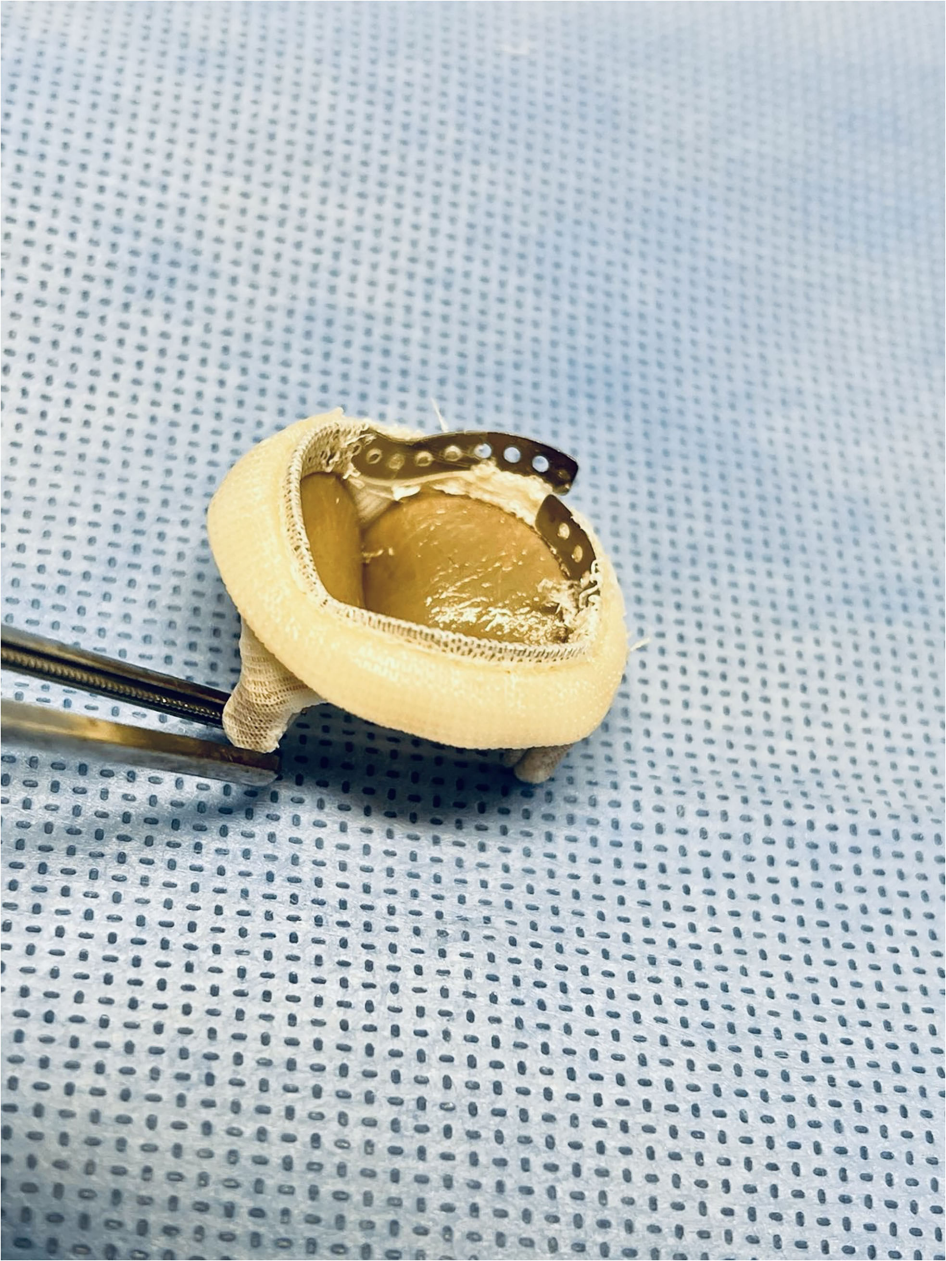
Visual confirmation of bioprosthetic valve fracturing after removal of the sewing ring.

BVF was confirmed under fluoroscopy (Figure 2), with visual inspection after removal of the sewing ring (Figure 3). The ratio of balloon diameter to inner prosthetic diameter was calculated.

### Failure of High-Pressure Balloons

Balloon failures were attributed to either ruptures (Supplementary Video 2) or pinhole defects (Supplementary Video 3). In the latter events, inflated balloon volumes remained visibly stable, but further pressure increase failed due to inherent microlesions.

## Results

### *In vitro* Bioprosthetic Valve Fracturing

Both the 19- and 21-mm PERIMOUNT P2900 valves were amenable to *in vitro* BVF, requiring balloons 1 mm larger than labeled valve sizes for procedural success. Applied pressures of 19–20 atm were sufficient to fracture the 19-mm valve, whereas pressures of 22–25 atm were needed for the 21-mm valve (Table 1). Fluoroscopy confirmed frame dehiscence in all valves tested. Caliper measurements also indicated increases in inner diameters after BVF, relative to baseline determinations (19-mm valve: 17.5 mm→ 20 mm; 21-mm valve: 20 mm→ 21.5 mm).

**Table 1:** *In vitro* fracturing studies of PERIMOUNT model P2900 bioprosthetic valve.

PM 19 - I	TD			
	20 mm			
	19 atm			
PM 19 -II	TD			
	20 mm			
	19 atm			
PM 19 - III	TD	TD		
	20 mm	20 mm		
	19 atm	19 atm		
PM 19 - IV	AG	TD		
	18 mm	20 mm		
	30 atm	19 atm		
PM 21 - I	TD	TD	TD	AG
	21 mm	21 mm	22 mm	22 mm
	20 atm	25 atm	20 atm	25 atm
PM 21 - II	TD	TD	AG	
	21 mm	20 mm	22 mm	
	20 atm	18 atm	22 atm	
PM 21 - III	TD	AG		
	22 mm	22 mm		
	22 atm	25 atm		
PM 21 - IV	AG	AG		
	20 mm	22 mm		
	30 atm	22 atm		

*Color-coded outcomes of bioprosthetic valve fracturing attempts:*

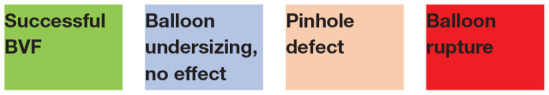

*TD, true dilatation balloon; AG, atlas gold balloon*.

### High-Pressure Balloon Performance

In the four 19-mm PERIMOUNT P2900 valves that were tested, BVF was consistently achieved using 20-mm True Dilatation Balloon Valvuloplasty Catheters at pressures of 19–20 atm (Table 1). One pinhole defect surfaced within this test series. An 18-mm Atlas Gold PTA Dilatation Catheter inflated to 30 atm remained intact but failed to achieve BVF.

Four 21-mm PERIMOUNT P2900 valves were similarly tested. BVF was consistently achieved using 22-mm Atlas Gold PTA Dilatation Catheters at pressures of 22–25 atm (Table 1). True Dilatation Balloon Valvuloplasty Catheters at 20-, 21-, and 22-mm sizes failed to achieve BVF due to ruptures or pinhole defects. A 20-mm Atlas Gold PTA Dilatation Catheter inflated to 30 atm remained intact but failed to achieve BVF.

### High-Pressure Balloon Defects

During *in vitro* BVF testing, the high-pressure balloons displayed two modes of failure. There were four ruptures of True Dilatation Balloon Valvuloplasty Catheters at pressures of 18–22 atm (mean, 20 atm), whereas all Atlas Gold PTA Dilatation Catheter remained intact. In the True Dilatation Balloon Valvuloplasty Catheters, pinhole defects undermined balloon pressurization, leading to three failed BVF attempts.

## Discussion

During *in vitro* testing of the Edwards PERIMOUNT P2900 valve (both 19- and 21-mm sizes), fracturing of its bioprosthetic ring was fully achievable. However, a balloon 1 mm larger than the labeled valve size (ie, 3 mm beyond inner prosthetic diameter) was required for success. In the 21-mm valve, higher pressure levels were required to achieve BVF. Only the Atlas Gold PTA Dilatation Catheter was capable of doing so, the True Dilatation Balloon Valvuloplasty Catheter failing entirely. Balloon failures resulted from true ruptures or pinhole defects.

### *In vitro* BVF Studies

Recent *in vitro* BVF studies have reported technical specifications and feasibility data for various surgical valves other than the PERIMOUNT P2900 (6, 7). Higher pressure levels (ie, 18–24 atm) were required for BVF of surgical bioprostheses with metal rings (e.g., Magna, Magna Ease [Edwards Lifesciences]), as opposed to those with polymer rings (e.g., Epic [Abbott Laboratories, Chicago, IL, USA], Mosaic [Medtronic, Dublin Ireland], Mitroflow [LivaNova, London, UK]) where 8–12 atm sufficed (5–7). Based on bench testing of analogous devices, balloon oversizing of 1 mm beyond stated valve dimension is recommended (6, 7). For the 19- and 21-mm PERIMOUNT Magna valves, the feasibility of BVF using either an Atlas Gold PTA Dilatation Catheter or a True Dilatation Balloon Valvuloplasty Catheter has been proven at high (24-atm) pressure levels (6). Although the PERIMOUNT P2900 and the Magna have similar fluoroscopic appearances, results of our *in vitro* test series differed. Pressure required (19–20 atm) for the 19-mm PERIMOUNT P2900 was lower than that required (24 atm) for the 19-mm Magna. Also, successful BVF of the 21-mm Magna has been reported at 24 atm, whether by Atlas Gold PTA Dilatation Catheter or True Dilatation Balloon Valvuloplasty Catheter (6). We did not achieve BVF in 21-mm P2900 valves using True Dilatation Balloon Valvuloplasty Catheters. Table 2 summarizes the currently available data on *in-vitro* BVF studies.

**Table 2:** Overview of perviously reported data and data acquired within the present study on required pressure rates to achieve *in-vitro* BVF using the atlas gold PTA dilatation catheter or true dilatation balloon valvuloplasty catheter (6, 7).

	**Labeled valve size**	**Atlas gold (mm)**	**Fracture pressure (atm)**	**True dilatation (mm)**	**Fracture pressure (atm)**	**Source data reference**
Perimount P 2900	19	20	Not tested	20	19	
Perimount P 2900	21	22	22–25	22	Failed	
Magna ease	19	20	19		Not tested	(7)
Magna ease	19	20	18	20	18	(6)
Magna ease	21	22	21		Not tested	(7)
Magna ease	21	22	18	22	18	(6)
Magna	19	20	24	20	24	(6)
Magna	21	22	24	22	24	(6)
Mosaic	19	20	10		Not tested	(7)
Mosaic	19	20	10	20	10	(6)
Mosaic	21	22	8		Not tested	(7)
Mosaic	21	20	10	20	10	(6)
Mitroflow	19	20	12	20	12	(6)
Mitroflow	21	22	10		Not tested	(7)
Mitroflow	21	22	12	22	12	(6)
St. Jude Epic	21	22	8	22	8	(6)

*orange indicated the surgical valves tested in the current study. gray performance of the Atlas Gold balloon in in-vitro BVF studies. blue performance of the True dilatation balloon in in-vitro BVF studies*.

### Causes of BVF Failure

Existing clinical data on BVF failure rates are sparse (9). Although balloon ruptures during ViV-TAVR procedures are quite evident by fluoroscopy, pinhole balloon defects are more likely signaled indirectly. For instance, manometer readings may indicate pressure loss or stagnation during full fluoroscopic balloon inflation. In such circumstances, balloons should be deflated, and attempts at BVF terminated. Balloon undersizing also precludes successful BVF.

### Clinical Data on BVF

Some clinical case series addressing ViV-TAVR have demonstrated lower transvalvular gradients through BVF, compared with its non-use or with postdilatation, respectively (4, 9). As defined by the Valve Academic Research Consortium (VARC), device success is reportedly higher after ViV-TAVR procedures if BVF is performed (93 vs. 68%; *p* < 0.001) (4); and transvalvular gradients seem to be lower (10). Midterm data on ViV-TAVR with BVF are scant. Immediate postoperative transvalvular gradients in 139 patients treated thusly were low (9.4 ± 5.8 mmHg) but increased significantly (14.6 ± 7.5 mmHg; *p* < 0.001) at 30 days and remained stable for up to 1-year of follow-up (11). BVF-related complications, such as stroke, annular rupture, and coronary obstruction, have been rare, not exceeding rates cited for ViV-TAVR only (9, 12).

### Technical Specifications for BVF Procedure

The present study provides technical specifications for BVF of PERIMOUNT P2900 bioprosthetic valves. Models P2900 and P2800 differ only in their pericardial leaflet treatments. Hence, we presume that the findings herein are applicable to the P2800 model as well.

We do suggest that balloons 1 mm larger than labeled valves be applied in this setting. *In vitro* BVF of the 21-mm PERIMOUNT P2900 also requires use of an Atlas Gold PTA Dilatation Catheter. Because pinhole defects seemed to account for nearly one-half of our balloon failures, we advise continuous monitoring of manometers during any BVF attempts to clearly identify such defects and abort all non-productive efforts.

Randomized clinical studies of ViV-TAVR procedures conducted alone or in conjunction with BVF are needed to verify the hemodynamic benefit of BVF and to establish protocols for its utilization.

## Conclusion

Both 19- and 21-mm PERIMOUNT P2900 valves are amenable to BVF, thereby increasing inner prosthetic diameters. High-pressure balloons 1 mm larger than labeled valve sizes are essential for this purpose, and the Atlas Gold PTA Dilatation Catheter alone should ensure success in 21-mm prosthetics.

## Limitations

Despite the limited number of valves tested, results were quite consistent. Still, an *in vitro* study may not entirely simulate *in vivo* BVF during transcatheter replacement of a degenerated surgical valve.

## Data Availability Statement

The raw data supporting the conclusions of this article will be made available by the authors, without undue reservation.

## Author Contributions

HR is responsible for the study design, data collection, data analysis, data interpretation, and writing the manuscript. HA-C, OD, and ZA contributed in the *in-vitro* testing and manuscript revision. KV revised the manuscript. RL revised the manuscript and supervised the project. All authors contributed to the article and approved the submitted version.

## Conflict of Interest

The authors declare that the research was conducted in the absence of any commercial or financial relationships that could be construed as a potential conflict of interest.

## Publisher's Note

All claims expressed in this article are solely those of the authors and do not necessarily represent those of their affiliated organizations, or those of the publisher, the editors and the reviewers. Any product that may be evaluated in this article, or claim that may be made by its manufacturer, is not guaranteed or endorsed by the publisher.

## Supplementary Material

Supplementary Video 1 Successful bioprosthetic valve fracturing.

Supplementary Video 2 High-pressure balloon rupture causing failure of bioprosthetic valve fracturing attempt.

Supplementary Video 3 With pressure increase microlesion with volume loss undermine bioprosthetic valve fracturing attempt.
